# Case report: Long term remission of metastatic sinonasal NUT carcinoma after palliative radiotherapy and immunotherapy in an elderly patient

**DOI:** 10.3389/fonc.2024.1412070

**Published:** 2025-01-07

**Authors:** Justin K. W. Ng, Edwin C. Y. Wong, Tommy C. Y. So, Raiden T. S. Wong

**Affiliations:** ^1^ Department of Clinical Oncology, Pamela Youde Nethersole Eastern Hospital, Hong Kong, Hong Kong SAR, China; ^2^ Department of Clinical Pathology, Pamela Youde Nethersole Eastern Hospital, Hong Kong, Hong Kong SAR, China

**Keywords:** sinonasal malignances, NUT carcinoma (NC), radiotherapy, immunotherapy, NUTM1 gene rearrangement

## Abstract

NUT carcinoma (NC) is an extremely rare, aggressive malignancy characterized by chromosomal rearrangements in the *NUTM1* (nuclear protein in testis) gene. It usually affects younger patients with a median age of diagnosis at 23 years old. The mainstay of treatment consists of combination chemotherapy, surgical resection, and high dose radiation. However, prognosis remains dismal with reported median overall survival of 6.7 months. Literature reporting on use of immunotherapy in head and neck NC is limited. Prolonged remission without aggressive multimodality therapy is rare. We report a case of a 87-year-old woman with metastatic sinonasal NC treated with palliative radiotherapy and pembrolizumab who achieved sustained response 2 years from diagnosis.

## Case presentation

An 87-year-old woman presented to our hospital in December 2021 with 2 months’ history of an enlarging right upper alveolar mass and facial numbness. Apart from history of osteoporosis and lacunar infarct on aspirin, she had no other significant medical comorbidities and was a lifelong nonsmoker.

Clinical examination revealed a 2 cm fleshy tumor over the right upper alveolus and mild bulging over the right maxillary region. There was no clinical evidence of overlying skin infiltration. Cranial nerves were grossly intact except there was decreased sensation over right V2 dermatome. There were no palpable cervical lymph nodes. ECOG performance status was 2.

**Figure 1:**
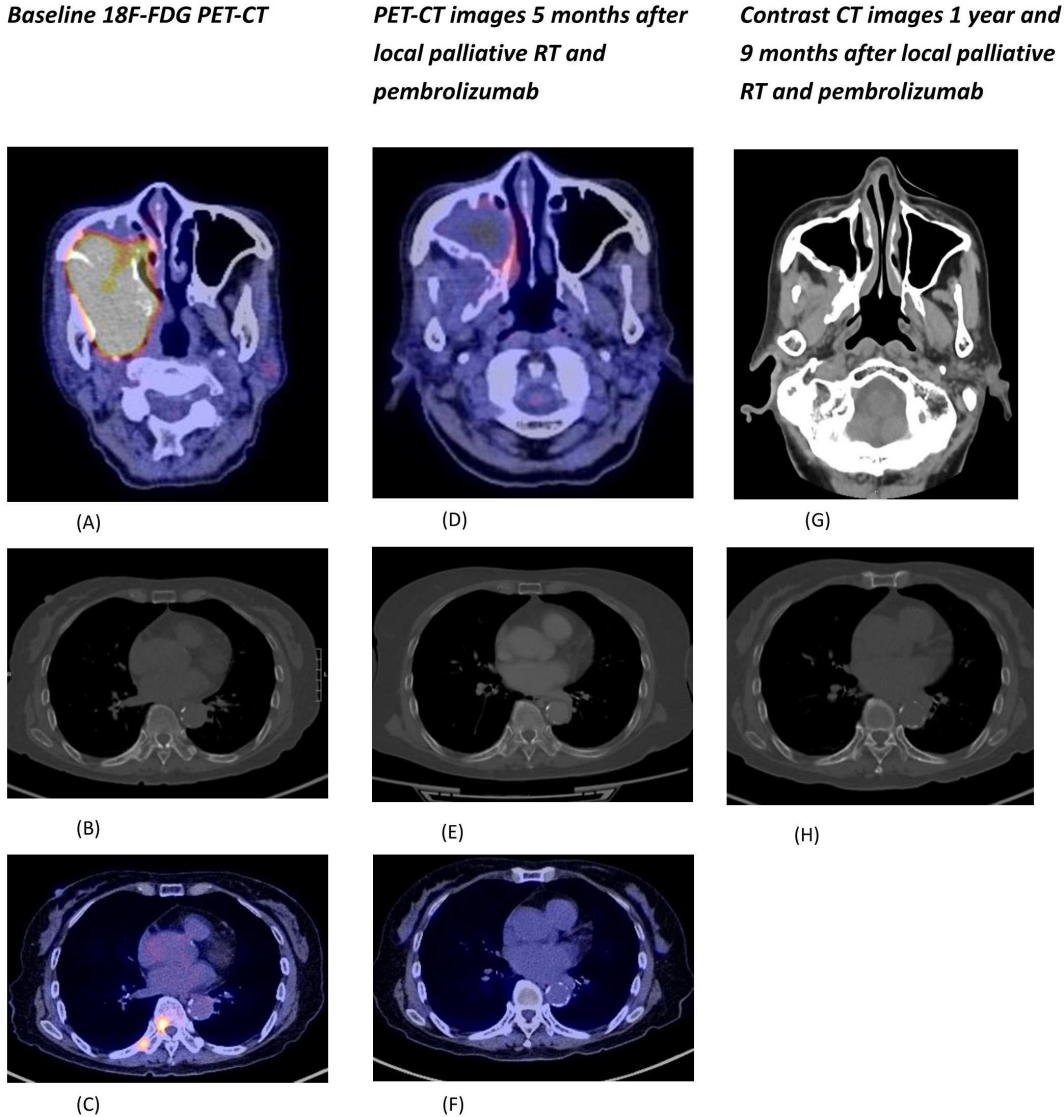
**(A)** Fusion PET-CT of the hypermetabolic primary tumor involving the right maxillary sinus and right parapharyngeal region. **(B)** CT (bone window) showing sclerotic bone metastases at right T9 and right 9th rib. **(C)** Fusion PET-CT of hypermetabolic bone metastases at right T9 and right 9th rib. **(D)** Fusion PET-CT of the primary tumor showing significant shrinkage and metabolic response. **(E)** CT (bone window) of lesions at right T9 and right 9th rib with radiological improvement. **(F)** Fusion PET-CT of lesions at right T9 and right 9th rib with metabolic response. **(G)** Contrast CT (soft tissue window) showing sustained tumor response with only residual mucosal thickening at the posterior wall of right maxillary sinus. **(H)** CT (bone window) of lesions at right T9 and right 9th rib with further radiological improvement.

Whole Body Positron Emission Tomography (18 F FDG) showed a large hypermetabolic mass at the right maxillary sinus measuring 8 x 6.2cm with extension to the right orbit, parapharyngeal space, pterygoid muscles and upper molar regions (**Figure 1A**) and multiple hypermetabolic osseous metastases (**Figures 1B, C**).

**Figure 2:**
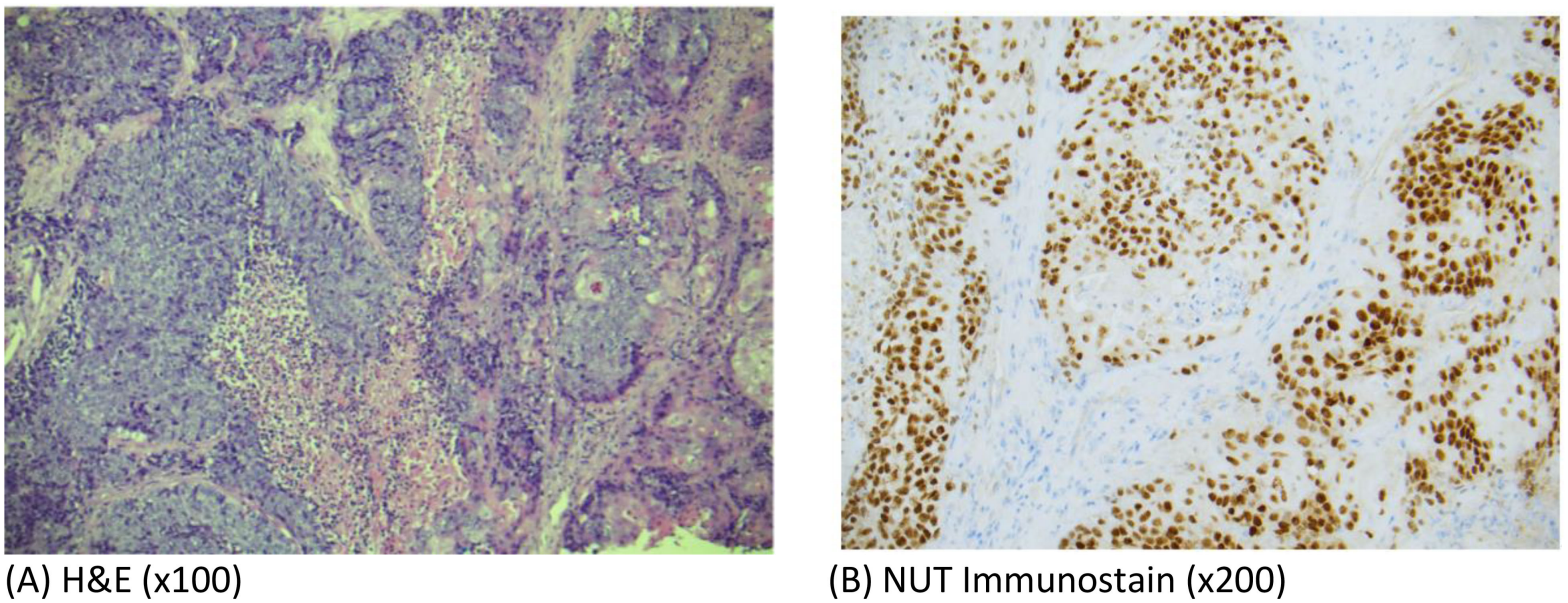
**(A)** Photomicrographs of hematoxylin-and-eosin (H&E)-stained sections (x100) from the tumor biopsy specimen showing sheets of malignant cells with high N/C ratio, mitotic activity and areas of abrupt keratinization consistent with poorly differentiated carcinoma. **(B)** Photomicrographs showing diffuse positivity of tumor cells with NUT immunostain (x200).

Incisional biopsy of the mass revealed malignant cells exhibiting high N/C ratio, mitotic activity, necrosis with areas with abrupt keratinization. Immunohistochemical staining of the malignant cells were diffusely positive for p40 and NUT immunostains (**Figure 2**). The final pathological diagnosis was NC. PDL-1 CPS score was 3 and TPS score was 2.

She was given palliative external beam radiotherapy 20Gy over 5 fractions to the maxilla tumor which was well tolerated with good clinical response, and followed by immunotherapy pembrolizumab (200mg IV Q3week). After one cycle of pembrolizumab, she developed derangement of liver function (ALT 335 ALP 336 total bilirubin 14). Baseline liver functions were all normal. Ultrasound of the hepatobiliary system showed no liver lesions. Liver function spontaneously normalized after 6 weeks of close observation without any additional medical treatment. Due to the occurrence of grade 2 immunotherapy related hepatitis, the patient opted not to resume further immunotherapy PET-CT scan 2 months after pembrolizumab administration showed significant shrinkage of the maxilla mass and both the primary and metastatic lesions became isometabolic (**Figures 1D–F**).

**Figure 3:**
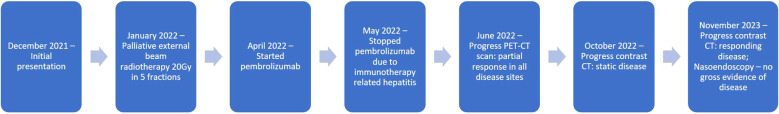
Treatment course of our patient.

Contrast CT in November 2023 showed responding disease with non-enhancing soft tissue thickening at the right maxillary sinus wall and continuous radiological improvement in the bone metastases (**Figures 1G, H**). Nasal endoscopy in November 2023 showed no definite exophytic lesion in the maxillary sinuses. In her last clinic visit in September 2024 (33 months from initial diagnosis), she reported no active symptoms from her malignancy. A summary of our patient's treatment course is provided in **Figure 3**.

## Discussion

First described in the 1990s, NUT carcinomas are poorly differentiated carcinomas defined by the presence of chromosomal rearrangements in the *NUTM1* gene on chromosome 15q14. The most common rearrangement is the t(15:19) translocation with the *BRD4* gene, accounting for around 67% of cases; less common alterations include *BRD3::NUTM1* or *NSD3::NUTM1* fusions. *BRD::NUTM1* fusions are critical to the pathogenesis of NC; their presence alone has been shown to be sufficient to be sufficient to drive malignant transformation (1). The fusion proteins are hypothesized to cause carcinogenesis by blocking cellular differentiation via interference with several key genetic targets, including *MYC, SOX2, MED24*, and *TP63* genes (2).

NC may arise from diverse organs, and most frequently originate from midline structures such as the sinonasal tract, lung, and thymus. Treatment pathways for NC usually follow that of the primary site. In head and neck NC, this consists of aggressive surgery and radiation for resectable cases. For unresectable or metastatic disease, the mainstay of treatment is combination platinum or ifosphamide based chemotherapy followed by surgical debulking and/or consolidation radiation.

Despite use of aggressive multimodality treatment regimens, the prognosis of head and neck NC remain poor. In two largest retrospective series conducted on head and neck NC (3, 4), median overall survival ranged from 9.7 to 14.6 months, and no patients with metastatic disease were reported surviving beyond two years.

Our patient, aged 87 at presentation, represents the oldest reported case of NC in the literature to date (5). Unlike younger NC patients, she was not a candidate for aggressive treatment, thus immunotherapy with immune checkpoint inhibitors (ICI) was considered.

Pembrolizumab has been approved for use in advanced HNSCC with high PDL 1 expression and confers long term overall survival benefit (6). In primary pulmonary NC, numerous case reports have shown encouraging responses to PD-1 inhibitors in pretreated disease (7, 8). Reported progression free survival ranged from 5 to 29 months and overall survival 19.5 to 79 months.

On the other hand, literature reporting on ICI use in head and neck NC is limited. There are 4 reported cases of ICI use in head and neck NC to date (see **Table 1**). The best responder was a 34-year-old male presenting with locally advanced NC of thyroid primary, with no evidence of disease 38 months from diagnosis. However, he underwent radical surgery and pembrolizumab given as adjuvant treatment. For cases with unresectable/metastatic disease, the best response occurred using nivolumab and radiotherapy to 70Gy, where 14 months disease control on ICI was achieved before further systemic progression. To our knowledge, our patient, in remission more than 2 years from diagnosis, has the longest disease control to date with ICI in metastatic head and neck NC.

**Table 1:** Case reports of head and neck NC treated with immunotherapy.

Author/Reference	Gender/Age	Disease site(s)	NUTM1 fusion, PDL-1 TMB	Duration of response with ICI	Overall survival	ICI used and treatment line
Herbison et al (9)	27, F	Unresectable disease, left nasal cavity. No distant metastases	BRD3 PDL-1 TPS < 1%	OligoPD at 7 months with SBRT given. Systemic PD after total 14 months	46 months, in remission	1^st^ line Docetaxel, cisplatin 5FU 2^nd^ line nivolumab concurrent RT 70Gy 3^rd^ line BETi
Caner et al (10)	29, F	Right nasal cavity, multiple bone metastases	BRD4 PDL-1 TPS 10%	No response, PD at 4 months	5.4 months, Died of PD	1^st^ line Cisplatin 5FU, Pembrolizumab. Addition of docetaxel, debulking surgery and RT after 1^st^ cycle due to inadequate response
Wei-Ning et al (11)	34, F	Parotid. Local and metastatic recurrence to bones and skull base	not provided	No response	6 months, died of PD	Initial localized disease treated with radical surgery and adjuvant RT Metastatic recurrence given 1^st^ line pembrolizumab and palliative RT to head and neck, spine.
Fu et al (12)	36, M	Parotid. Metastatic recurrence to liver, lymph nodes	BRD4 PDL-1 TPS and CPS < 1% TMB 4Mut/Mb	5 months, partial response	24 months, died of PD	Initial localized disease treated with radical surgery and adjuvant RT, chemotherapy and cetuximab. Metastatic recurrence with multiple lines of treatment (RFA, VAC/IE, BETi) Sintilimab + Lenvatinib as 4^th^ line.
Kuo et al (13)	34, M	Thyroid, Locally advanced disease. No distant metastases	NSD3	38 months, in remission	-	Radical surgery, post op chemoRT 66Gy with cisplatin Adjuvant pembrolizumab for 18 months.

Prior studies suggest NCs are immunologically cold tumors with low PDL-1 expression and tumor mutational burden (14). Our patient had PDL-1 TPS and CPS scores of 2 and 3 respectively, which predicts poor response to ICI monotherapy. Yet, prolonged disease control was achieved with pembrolizumab. Since ICI administration was preceded by palliative radiotherapy, radiation may have enhanced the immunotherapy response. This is supported by preclinical studies demonstrating radiation exerts a synergistic effect with immunotherapy via facilitating tumor associated antigen release, upregulating PDL-1 expression on tumor cells and increasing T cell infiltration (15, 16).

In non-small cell lung carcinoma (NSCLC), a phase II trial of pembrolizumab and stereotactic body radiotherapy doubled overall response rate compared to ICI alone, mainly in PDL-1 negative tumors; suggesting radiotherapy may activate non inflamed tumors to an inflamed microenvironment responsive to ICI (17). In pulmonary NC, a review of 12 cases treated with ICI revealed better outcomes in patients who also received radiation (18). The promising outcomes achieved with radiation and immunotherapy suggests this combination warrants further study in clinical trials for NC. Data on expression of immune markers (such as tumor infiltrating lymphocytes and circulating immune cells) before and after radiotherapy would be particularly valuable to delineate the role of radiation and immunotherapy for this disease.

In the 4 cases of head and neck NC identified in the literature, (**Table 1**) all received radiotherapy, yet outcomes were heterogenous. Thus, factors apart from radiation may have contributed to our patient’s exceptional response.

Despite our patient’s advanced age, she was in relatively fit medical condition. Her basic activities of daily living her largely independent and she had very few significant medical comorbidities. This may have been a contributing factor to the immune response achieved, Prior studies on ICI use in head and neck cancer patients have also shown better survival and tumor response rates in patients without comorbidities (19). Ethnicity may have also played a role, as Asian origin has recently been suggested in meta analyses to confer better survival outcomes with ICI (20).

Our patient had immunotherapy stopped after one cycle due to suspected immune related hepatitis; this may have predicted for a stronger antitumor effect from pembrolizumab. The occurrence of immune related adverse events is increasingly recognized as a clinical biomarker for ICI response (21). In NSCLC and melanoma occurrence of immune related adverse events is associated with favorable treatment response despite early cessation of ICI (22, 23).

The exact underlying mechanisms are unclear. Studies have suggested tumor and organs with subclinical inflammation share common antigens, resulting in a common T cell response at both sites (24). Other studies have linked gut microbiome composition to higher rates of tumor response and IO colitis (25).

Another factor is the type of *NUTM1* gene fusion, since studies have suggested atypical variant fusion partners (e.g *BRD3/NSD3*) carry better prognoses compared the commonest BRD4 translocation (26). Unfortunately, inadequate quality of the biopsy specimen prevented identification of gene fusion in our case.

Apart from immunotherapy, recent phase I/II clinical trials have tested use of BET inhibitors (BETi), which inhibits binding of *BRD* to chromatin, thereby disrupting activity of the *NUTM1* fusion protein. So far only modest results have been achieved with BETi monotherapy; one of the larger phase I trials comprising of 19 patients reported PR in 4 patients and median PFS of 2.5 months (27). Given the favorable responses that have been achieved with immunotherapy and radiation in our case, using ICI and/or radiation therapy combined with BETi may represent a worthwhile strategy for further research. Preclinical studies have demonstrated BET inhibitors may combine synergistically with PD-1 inhibitors by downregulating T cell PD 1 expression and remodeling immunosuppressive tumor microenvironments (28, 29). BETi has also been shown in prostate cancer xenograft models to enhance the efficacy of radiotherapy and overcome radioresistance (30). In the two case reports where BET inhibitors were used before and after anti PD -1 therapy respectively for head and neck NC, partial tumor responses were achieved (refer to **Table 1**). Further clinical trials are awaited to explore the efficacy of BETi, radiotherapy and ICI combinations.

## Conclusion

Although metastatic head and neck NC is characterized by poor prognosis, our case illustrates use of immunotherapy and radiation can produce a durable response. ICIs may still be considered in patients who are otherwise unfit for traditional aggressive multimodality treatments. More studies are warranted to explore the efficacy of immunotherapy and its potential interactions with other modalities such as radiation, chemotherapy and BET inhibition.

## Data Availability

The original contributions presented in the study are included in the article/supplementary material. Further inquiries can be directed to the corresponding author.
